# Strongyloidiasis and Infective Dermatitis Alter Human T Lymphotropic Virus-1 Clonality *in vivo*

**DOI:** 10.1371/journal.ppat.1003263

**Published:** 2013-04-04

**Authors:** Nicolas A. Gillet, Lucy Cook, Daniel J. Laydon, Carol Hlela, Kristien Verdonck, Carolina Alvarez, Eduardo Gotuzzo, Daniel Clark, Lourdes Farré, Achiléa Bittencourt, Becca Asquith, Graham P. Taylor, Charles R. M. Bangham

**Affiliations:** 1 Section of Immunology, Wright-Fleming Institute, Imperial College London, London, United Kingdom; 2 Molecular and Cellular Epigenetics, Interdisciplinary Cluster for Applied Genoproteomics (GIGA) of University of Liège (ULg), Liège, Belgium; 3 Instituto de Medicina Tropical Alexander von Humboldt and Hospital Nacional Cayetano Heredia, Universidad Peruana Cayetano Heredia, Lima, Peru; 4 Institute of Tropical Medicine, Antwerp, Belgium; 5 Laboratory of Experimental Pathology, Oswaldo Cruz Foundation, Salvador, Bahia, Brazil; 6 Complexo Hospitalar Universitário Prof. Edgard Santos, Department of Pathology, Federal University of Bahia, Salvador, Bahia, Brazil; 7 Section of Infectious Diseases, Wright-Fleming Institute, Imperial College London, London, United Kingdom; University of Medicine & Denistry New Jersey, United States of America

## Abstract

Human T-lymphotropic Virus-1 (HTLV-1) is a retrovirus that persists lifelong by driving clonal proliferation of infected T-cells. HTLV-1 causes a neuroinflammatory disease and adult T-cell leukemia/lymphoma. Strongyloidiasis, a gastrointestinal infection by the helminth *Strongyloides stercoralis*, and Infective Dermatitis associated with HTLV-1 (IDH), appear to be risk factors for the development of HTLV-1 related diseases. We used high-throughput sequencing to map and quantify the insertion sites of the provirus in order to monitor the clonality of the HTLV-1-infected T-cell population (i.e. the number of distinct clones and abundance of each clone). A newly developed biodiversity estimator called “DivE” was used to estimate the total number of clones in the blood. We found that the major determinant of proviral load in all subjects without leukemia/lymphoma was the total number of HTLV-1-infected clones. Nevertheless, the significantly higher proviral load in patients with strongyloidiasis or IDH was due to an increase in the mean clone abundance, not to an increase in the number of infected clones. These patients appear to be less capable of restricting clone abundance than those with HTLV-1 alone. In patients co-infected with *Strongyloides* there was an increased degree of oligoclonal expansion and a higher rate of turnover (i.e. appearance and disappearance) of HTLV-1-infected clones. In *Strongyloides* co-infected patients and those with IDH, proliferation of the most abundant HTLV-1^+^ T-cell clones is independent of the genomic environment of the provirus, in sharp contrast to patients with HTLV-1 infection alone. This implies that new selection forces are driving oligoclonal proliferation in *Strongyloides* co-infection and IDH. We conclude that strongyloidiasis and IDH increase the risk of development of HTLV-1-associated diseases by increasing the rate of infection of new clones and the abundance of existing HTLV-1^+^ clones.

## Author Summary

HTLV-1 is a human retrovirus estimated to infect 20 million people world-wide and is causing in a small proportion of the infected individuals an inflammatory disease or a leukemia/lymphoma. HTLV-1 persists lifelong by driving clonal proliferation of infected T-cells. Strongyloidiasis, a gastrointestinal infection by an helminth (*Strongyloides stercoralis*) and Infective Dermatitis associated with HTLV-1 (IDH), a skin inflammation with bacterial infection, appear to increase the risk of developing HTLV-1-related diseases. It is well known that the chance of developing HTLV-1-related diseases increases with the number of cells infected by the virus (also called proviral load). It is also known that HTLV-1-infected individuals co-infected by *Strongyloides* or affected by IDH have a higher proviral load, but the mechanism is still unclear. Consequently, the aim of this study was to test if co-infection increases the total number and/or the abundance (or size) of HTLV-1-infected T-cell clones. We have shown that the significantly increased proviral load in HTLV-1-infected individuals with IDH or strongyloidiasis is due to an increase in the mean clone abundance (bigger clones), not to an increase in the number of infected clones. These patients appear to be less capable of restricting clone abundance than those with HTLV-1 alone.

## Introduction

Human T-lymphotropic virus type 1 (HTLV-1) causes adult T-cell leukemia-lymphoma (ATLL) and HTLV-1-associated myelopathy/tropical spastic paraparesis (HAM/TSP). It has been estimated that 10 to 20 million persons live with HTLV-1 infection worldwide. A small proportion (up to 7%, depending on the area) of HTLV-1-infected individuals develop disease, while the majority remain asymptomatic carriers (ACs). Infection occurs via breastfeeding, transfusion of infected cellular blood products, or sexual intercourse. Symptoms usually appear after a long period (years or decades) of clinical latency [1]. The HTLV-1 proviral load remains stable within each infected person and correlates with the outcome of infection. However, the proviral load varies widely among infected people, even within a particular diagnostic group [2], [3], [4]. Infectious transmission of HTLV-1 across the virological synapse [5] is believed to be important early in infection, whereas mitotic replication is thought to be mainly responsible for maintaining proviral load once a persistent infection has been established and has reached equilibrium with the immune response [6]. We recently showed that the abundance of each established HTLV-1 clones is determined by genomic features of the host DNA flanking the provirus. HTLV-1 clonal expansion in vivo is enhanced by proviral integration in an actively transcribed area of the genome [7].

The helminth *Strongyloides stercoralis* (St) is estimated to infect 50-100 million individuals, mainly in the tropics and subtropics. Most people with strongyloidiasis have mild diarrhea, vague abdominal complaints, pruritus, perianal rash or simply remain asymptomatic. The *Strongyloides stercoralis* larvae migrate to a range of sites like the lung, liver, kidney, and central nervous system. The larvae can carry bacteria from the colon and cause fatal sepsis and meningitis. A severe form of the disease named strongyloides hyperinfection syndrome, characterized by abundant disseminated parasites, has been described in patients with corticosteroid therapy, severe malnutrition, transplantation, haematological malignancies (especially lymphoma) and HTLV-1 infection [8]. Epidemiological evidence shows that HTLV-1 is associated with a high frequency of *Strongyloides stercoralis* infection, a high risk of the strongyloides hyperinfection syndrome, and with relapse after treatment with ivermectin, thiabendazole, or albendazole [9], [10], [11], [12], [13], [14]. Patients with HTLV-1 and *Strongyloides stercoralis* co-infection had a higher HTLV-1 proviral load and a higher *Strongyloides stercoralis* burden than patients with either infection alone [13], [15]. The high proviral load measured in *Strongyloides stercoralis* co-infected patients has been linked with oligoclonal expansion of HTLV-1 infected T-cells [16]. *Strongyloides stercoralis* co-infection is suspected to be a risk factor for the development of ATLL, but the mechanism is still unclear [17], [18], [19], [20], [21], [22], [23], [24].

Infective dermatitis (IDH) is a severe, chronic, relapsing dermatitis associated with HTLV-1. IDH has been reported in several populations with endemic HTLV-1 infection, including South Africa, Jamaica, Trinidad, Brazil, Colombia, Peru and Japan. *Staphylococcus aureus* and/or *beta-hemolytic Streptococci* are commonly cultured from the skin lesions. The average age at disease onset is 2 years. The disease decreases in severity with age and rarely continues until adulthood [25]. IDH patients typically have a high HTLV-1 proviral load, comparable to HAM/TSP patients [26]. IDH is suspected to increase the risk of HAM/TSP or ATLL development [26], [27], [28], [29], but the evidence is not conclusive.

The aim of this study was to identify and quantify the impact of co-infection on HTLV-1 clonality. Because HTLV-1 proviral load and oligoclonality are closely correlated with disease status, we aimed to test the hypothesis that each co-infection increases the risk of HTLV-1-associated diseases by increasing the number or the abundance of HTLV-1-infected T-cell clones. We used a newly developed method to map and quantify thousands of HTLV-1 proviral insertion sites. We demonstrate that co-infections significantly alter the HTLV-1 clonality. Patients with strongyloidiasis or IDH had a higher proviral load and a higher average clone abundance than did asymptomatic HTLV-1 carriers even though the major determinant of proviral load was still the number of clones. The degree of oligoclonality of HTLV-1 was higher, and less stable over time, in patients with strongyloidiasis than in patients with neither co-infection nor ATLL, and there was a higher rate of turnover (i.e. appearance or disappearance) of HTLV-1-infected clones in the co-infected patients. Finally, we show that co-infections drive the proliferation of HTLV-1^+^ T-cell clones regardless of the genomic environment of the provirus, in contrast to infection with HTLV-1 alone, in which selective clonal expansion is favored by specific features of the proviral integration site in that clone.

## Results

### Co-infections alter HTLV-1 proviral load, average clone abundance and clonal distribution

**Figure 1:**
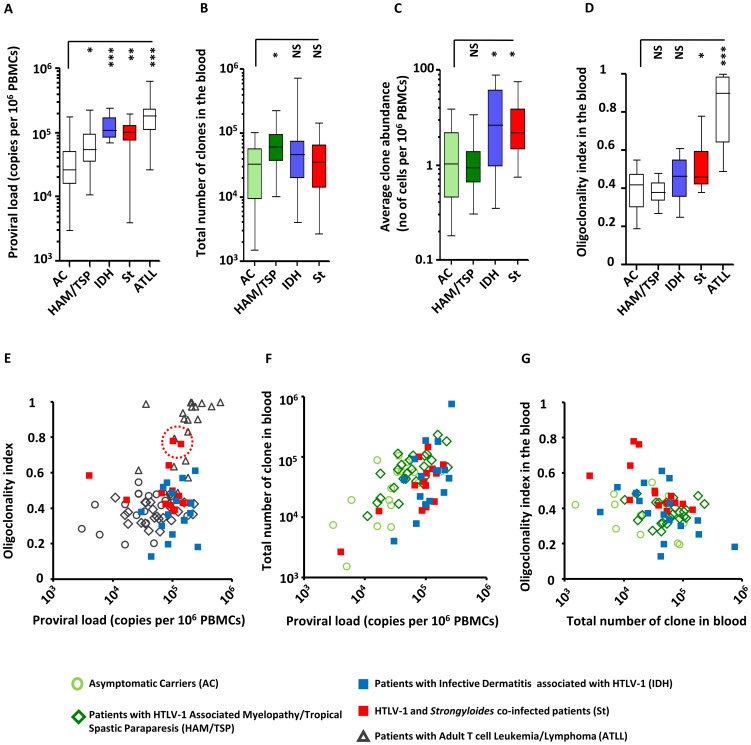
HTLV-1 clonal structure in the blood of subjects with HTLV-1 infection alone and those with co-infections. **A.** The mean proviral load of patients with IDH or *Strongyloides* co-infection was higher than the proviral load of asymptomatic carriers (ACs) (Mann Whitney, respectively p = 0.0001 and p = 0.002). **B.** The estimated total number of clones in the blood of co-infected patients was comparable to the total number of clones of ACs (Mann Whitney, respectively for IDH and *Strongyloides* co-infected vs AC, p = 0.27 and p = 0.81). By contrast, the estimated total number of clones in the blood of HAM-TSP patients was higher than those of ACs (Mann Whitney, p = 0.03) **C.** On average, the infected clones in the blood of IDH and *Strongyloides* co-infected patients were more abundant than those in ACs (Mann Whitney, respectively for IDH and *Strongyloides* co-infected vs AC, p = 0.02, and p = 0.02) and those in HAM/TSP patients (Mann Whitney, respectively for IDH and St vs HAM/TSP, p = 0.006, and p = 0.002). **D.** The oligoclonality index in the blood of IDH patients was not significantly different from that in ACs (Mann Whitney, p = 0.08). On the contrary, the oligoclonality index in peripheral blood of *Strongyloides* co-infected patients was higher than that in ACs (Mann Whitney, p = 0.01). **E.** proviral load and oligoclonality index were not correlated in any of the cohorts except ATLL (Spearman, respectively for AC, HAM/TSP, IDH, *Strongyloides* co-infected and ATLL, p = 0.34, p = 0.27, p = 0.92, p = 0.58 and p = 0.004). **F.** Proviral load was correlated with the total number of clones in the blood in each cohort (Spearman, respectively for AC, HAM-TSP, IDH and St, p = 0.02, p = 0.0008, p = 0.05 and p = 0.04). **G.** oligoclonality index and total number of clones in the blood did not correlate in any cohorts (Spearman, respectively for AC, HAM-TSP, IDH and St, p = 0.46, p = 0.27, p = 0.64 and p = 0.58). In panels A, D and E, data on patients infected with HTLV-1 infection alone are shown for comparison purposes and were originally published in *Blood*. Gillet *et al.* The host genomic environment of the provirus determines the abundance of HTLV-1-infected T-cell clones. Blood. 2011; 117: 3113–3122. These data are shown by empty whiskers or grey symbols to distinguish them from new data in color.

The proviral load in patients with IDH and in patients co-infected with *Strongyloides* was significantly higher than the proviral load in asymptomatic carriers (Figure 1A, median proviral load = 0.3*10^5^ proviral copies per 10^6^ PBMCs for Asymptomatic Carriers, 1.1*10^5^ for patients with Infective Dermatitis associated with HTLV-1 and 1.0*10^5^ for patients co-infected with *Strongyloides*, Mann Whitney, p = 0.0001 and p = 0.002 respectively for IDH and *Strongyloides* co-infected patients vs Asymptomatic Carriers). These observations are in accordance with data from previous reports [26], [28], [30]. We estimated the total number of clones present in the blood using a newly developed method (DL, BA, CRMB, submitted). We found that the total number of clones in the blood of IDH and *Strongyloides* co-infected patients was comparable to those of asymptomatic carriers (Figure 1B, Mann Whitney, respectively p = 0.27 and p = 0.81). We also observed that HAM/TSP patients had a higher number of clones in the blood than asymptomatic carriers (Figure 1B, Mann Whitney, p = 0.05). The average clone abundance (expressed as the number of cells in a given clone per 10^6^ PBMCs) was higher in patients with IDH and *Strongyloides* co-infection than in asymptomatic carriers, but no significant difference was observed between asymptomatic carriers and HAM/TSP patients (Figure 1C). The mean clone abundance, expressed as number of cells per 10^6^ PBMCs, was 1.4 in Asymptomatic Carriers, 1.2 (HAM/TSP), 3.6 (IDH patients) and 3.0 (S*trongyloides* co-infected patients) (Figure 1C, Mann Whitney, respectively for HAM/TSP, IDH and *Strongyloides* vs Asymptomatic Carriers, p = 0.97, p = 0.02 and p = 0.02). The distribution of clone abundance is depicted in Figure S1A in Text S1. Each slice in the pie charts represents a single clone; the size of the slice is proportional to the relative abundance of that clone. The 3 most abundant clones are represented by the colored slices. The clonality of HTLV-1 in the blood of the representative patient with IDH was relatively uniform (the slices in the pie charts are of similar size) and accordingly the oligoclonality index was low in this representative subject (Figure S1A in Text S1). The HTLV-1 clone distributions in two different patients co-infected with *Strongyloides stercoralis* are shown to illustrate the wide variation in clonality observed in this group of patients despite similar proviral load. The oligoclonality index of the first patient was low and in the range observed in Asymptomatic Carriers and patients with HAM/TSP. The oligoclonality index of the second patient with *Strongyloides* was greater because of the presence of 3 relatively abundant clones (Figure S1A in Text S1, red slices made up nearly half of the proviral load) and lay in the range of patients with ATLL (Figure 1E, red dotted circle). The oligoclonality index in patients with *Strongyloides* co-infection was greater than the oligoclonality index in Asymptomatic Carriers (Figure 1D, Mann Whitney, p = 0.01) confirming the previous observation of oligoclonal expansion of HTLV-1 infected T-cells [16]. There was no significant difference in oligoclonality index between Asymptomatic Carriers and IDH patients (Figure 1D, Mann Whitney, p = 0.10). Two patients out of the 14 co-infected with *Strongyloides* had a very high oligoclonality index (0.76 and 0.78 respectively) due to oligoclonal expansion of infected clones (Figure 1E, dotted red circle). Their proviral load and clonal distribution were in the range of patients with ATLL. In patients without malignant disease, proviral load did not correlate with oligoclonality index (Figure 1E). However, proviral load was positively correlated with the total number of clones in each cohort (Figure 1F) and by contrast no correlation was observed between the number of clones and oligoclonality index in any cohort (Figure 1G). The mean oligoclonality index in patients with *Strongyloides* did not vary significantly after anti-helminth treatment (Figure S1B and Table S2 in Text S1), although there was a large decrease in oligoclonality index after *Strongyloides* clearance in the patient with the most oligoclonal distribution in the cohort (Figure S1B in Text S1, patient St6).

These data show that, in individuals without ATLL, strongyloidiasis or IDH, the proviral load of HTLV-1, which correlates with the risk of inflammatory and malignant diseases, is determined mainly by the number of infected T-cell clones and not, as previously believed, by the amount of oligoclonal proliferation. The significantly increased proviral load in HTLV-1-infected individuals with IDH or co-infected with *Strongyloides* is due to an increase in the mean clone abundance, not to a further increase in the number of infected clones. Moreover, the degree of oligoclonal expansion in patients with *Strongyloides* was significantly higher than that in asymptomatic carriers, whereas no significant difference in oligoclonality index was observed between IDH patients and asymptomatic carriers.

### HTLV-1 clonality is less stable over time in *Strongyloides stercoralis* co-infected patients

**Figure 2:**
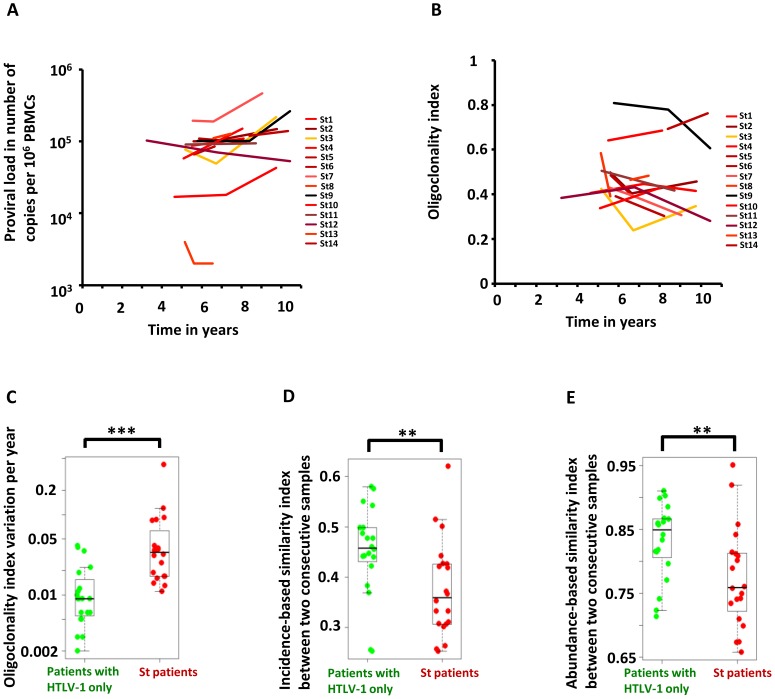
Temporal evolution of HTLV-1 proviral load and clonality in individuals with HTLV-1 infection alone or those with *Strongyloides* co-infection. **A.** Proviral load in HTLV-1 patients with *Strongyloides stercoralis* co-infection. **B.** Oligoclonality index in HTLV-1 patients with *Strongyloides stercoralis* co-infection. **C.** Variation in oligoclonality index during follow-up was significantly greater within co-infected patients than in patients with HTLV-1 only (Mann Whitney; p<0.0001). **D. and E.** HTLV-1 clone populations in two consecutive blood samples in *Strongyloides* co-infected patients differed more in identity and abundance than the clone populations from two consecutive blood samples in patients with HTLV-1 only (Mann Whitney; p = 0.007 and p = 0.007 respectively for incidence- and abundance-based similarity index).

Figure 2A shows the evolution of proviral load with time in *Strongyloides* co-infected patients. Figure 2B shows the evolution of oligoclonality index with time in *Strongyloides* co-infected patients. The data show that the oligoclonality index in *Strongyloides* co-infected patients was more variable over time than in patients with HTLV-1 infection alone as described previously [7]. This conclusion was confirmed by the data shown in Figure 2C, which depicts the absolute variation in oligoclonality index per year. The oligoclonality index varied on average by 0.01 per year in patients with HTLV-1 only and by 0.04 per year in *Strongyloides* co-infected patients (Mann Whitney, p<0.0001). The similarity between the populations of HTLV-1-infected T-cell clones at two consecutive time points is shown in Figure 2D and 2E. To compare these two populations we used the SØrensen similarity indices, which were developed to assess the similarity between two ecosystems in term of species shared. A clone is here the equivalent of a species. The indices range from 0 to 1, with 0 indicating that no clones are shared between the two time points and 1 indicating complete identity (see Materials and Methods). Figure S2 in Text S1 shows the incidence- and abundance-based similarity indices from biological replicates (i.e. clonality analyses made in triplicate from the same blood sample of patients with HTLV-1 only with non-malignant infection). The results show that HTLV-1 clone populations in two consecutive blood samples in *Strongyloides* co-infected patients differed more in identity and abundance than the clone populations from two consecutive blood samples in patients with HTLV-1 only (Figure 2D and 2E, Mann Whitney; p = 0.007 and p = 0.007 respectively for incidence- and abundance-based similarity index).

We conclude that HTLV-1 clonality was less stable in *Strongyloides* co-infected patients, in whom there was a higher rate of turnover of clones. This observation raised the question: does co-infection with *Strongyloides* alter the selection forces that favor selective expansion of HTLV-1^+^ clones?

### Co-infections drive the proliferation of clones regardless of the genomic environment of the provirus

**Figure 3:**
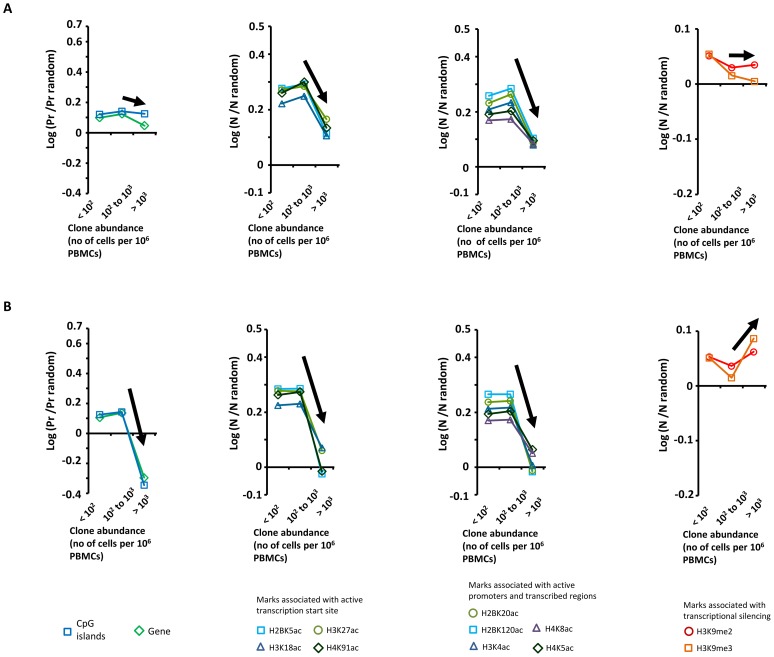
Genetic and epigenetic environment around the proviral insertion site. Proviral insertion sites identified in vivo were analysed according to disease status (patients co-infected with *Strongyloides stercoralis* in panel **A** and patients with infective dermatitis in panel **B**) and clone abundance (number of cells in given clone per 10^6^ PBMCs). The y-axis shows the departure from the random distribution. “Pr” is the proportion of insertion sites lying within +/−10 kb of a CpG island or a RefSeq gene. Enrichment toward a given mark is calculated as log (Pr/Pr random), where Pr random is the proportion expected in perfect random integration. “N” is the number of a given epigenetic mark in a 10 kb window (+/−5 kb) around the insertion site. “N random” is the number of that mark in randomly distributed insertion sites. Enrichment of a given epigenetic mark was calculated as log (N/N random). In contrast with the trends observed in patients infected with HTLV-1 only [7], the abundance of the largest clones in patients with *Strongyloides stercoralis* or IDH was independent of the proviral insertion site environment. Sample size (n = number of proviral insertion sites) for each clone abundance category: “St, abundance <10^2^”, n = 24,110; “St, abundance between 10^2^ and 10^3^”, n = 2,480; “St, abundance >10^3^”, n = 47; “IDH, abundance <10^2^”, n = 18,726; “IDH, abundance between 10^2^ and 10^3^”, n = 2,265; “IDH, abundance >10^3^”, n = 31.

In patients with HTLV-1 alone, we previously reported [7] a positive correlation between clone abundance and proximity to CpG islands and host genes, a positive correlation between clone abundance and markers of active transcription, and a negative correlation with repressive epigenetic marks. We concluded from these data [7] that transcriptional activity of the flanking host genome favors selective expansion of the HTLV-1^+^ T-cell clone. In contrast, in patients with *Strongyloides* co-infection or IDH, we did not observe these trends linking clone abundance and a particular genomic environment of the proviral integration site (Figure 3, see black arrows). Specifically, the most abundant clones (clone having more than 10^3^ cells per 10^6^ PBMCs) had a genomic environment of the provirus similar to the random distribution; i.e. the environment of the provirus does not determine the abundance of the major clones in co-infected patients.

We conclude that the abundance of the largest clones in patients with *Strongyloides* co-infection or IDH is independent of the genomic environment of the proviral insertion site.

### Low oligoclonality index in the skin lesions from IDH patients

We quantified HTLV-1 clonality in two types of sample in which the infected T-cells present are believed to play a direct role in the pathogenesis of the respective inflammatory disease: CSF from patients with HAM/TSP and skin lesions from patients with IDH. The rationale was two-fold: first, over-representation of a few infected clones in these tissues may indicate immune activation of antigen-specific T-cells. Second, we wished to test whether the selectively expanded clones in these tissues are also abundant in the bloodstream.

**Figure 4:**
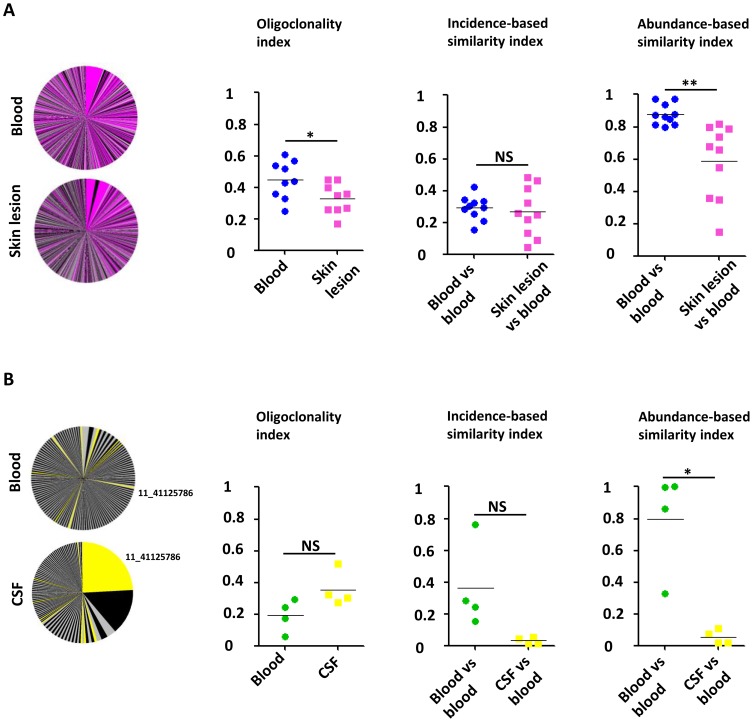
Comparison of the HTLV-1 clone populations between blood and skin lesions of IDH patients and between blood and CSF of HAM/TSP patients. **A.** Clonal distribution in the blood and the corresponding skin lesion of a representative patient with IDH. The pink slices denote clones present in both blood and skin lesion, the grey and black slices denote clones detected only in blood or skin. The mean oligoclonality index in the infected cell population in the skin lesion was significantly lower than the oligoclonality index in the corresponding blood sample (paired t-test, p = 0.015). The mean value of the incidence-based similarity index calculated between blood and skin populations was comparable to those between two blood subsamples (paired t-test, p = 0.646). By contrast, the mean value of the abundance-based similarity index calculated between blood and skin populations was significantly lower than those between two blood subsamples (paired t-test, p = 0.003). **B.** Clonal distribution in the blood and the corresponding CSF of a representative patient with HAM/TSP. Colored slices denote clones detected in both blood and CSF. The mean oligoclonality index in the CSF tended to be higher than the oligoclonality index in the blood, but the difference was not statistically significant (paired t-test, p = 0.214). The mean value of the abundance-based similarity index calculated between blood and CSF populations was significantly lower than those between two blood subsamples (paired t-test, p = 0.014). The mean value of the incidence-based similarity index calculated between blood and CSF populations was also lower than those between two blood subsamples, with marginal statistical significance (paired t-test, p = 0.090).

Figure 4A illustrates the overlap between the HTLV-1 infected cell populations from blood and from the skin lesion. The pink slices denote clones present in both blood and skin lesion, the grey and black slices denote clones detected only in blood or skin. The results show that a high proportion of the observed clones were present in both skin and blood samples. The infected cell population in the skin did not differ from the blood populations in the identity of the clones (incidence-based similarity index, paired t-test, p = 0.646), but differed by the relative abundance of the common clones (abundance-based similarity index, paired t-test, p = 0.003). The mean oligoclonality index in the infected cell population in the skin lesion was significantly lower than the oligoclonality index in the corresponding blood sample (paired t-test, p = 0.015). That is, all HTLV-1-infected T-cell clones present in the skin lesion had approximately the same relative abundance, and there was no evidence of selective expansion of a specific subset of infected cells.

Figure 4B illustrates the small overlap observed between blood and CSF HTLV-1 infected cell populations. As above the yellow slices show the clones found in both blood and CSF. The largest clone in the CSF contained a provirus inserted in chromosome 11 (coordinate 41125786): this clone was also detected in blood but at a much lower relative abundance. The infected clone population in the CSF differed in both identity and abundance from that in blood (incidence and abundance-based similarity index, paired t-test, respectively p = 0.090 and p = 0.014). In other words, the clone population in the CSF did not appear to be a random subsample of the blood population but diverged significantly, with the presence of other clones and a significant variation in the relative abundance of common clones. We conclude that there was selective migration or proliferation of infected T-cells in the CSF.

## Discussion

The data show that HTLV-1 clonality is extensively altered in vivo by *Strongyloides* co-infection and IDH. By comparison with patients infected with HTLV-1 only, *Strongyloidiasis stercoralis* co-infected patients showed five main differences: i. a higher mean proviral load, ii. a higher mean clone abundance, iii. a higher mean oligoclonality index, iv. a higher clone turnover rate (i.e. a higher rate of appearance and disappearance of clones), and v. a proliferation of the largest clones independent of the genomic environment of the provirus. IDH patients had also a higher mean proviral load, a higher mean clone abundance and a change in the selection forces that favor expansion of the largest HTLV-1 clones. The median oligoclonality index in the IDH group was higher than that in *Strongyloides* co-infected patients (respectively 0.464 vs 0.460), but the difference from asymptomatic carriers did not reach statistical significance. We emphasize that the mean age of the IDH cohort (14 years) was the lowest in the study (because the disease typically manifests during childhood); the mean age of *Strongyloides* co-infected patients was 44 years and that of asymptomatic carriers was 55 years. It would be interesting to follow the oligoclonality index over time in these IDH patients. The cohorts of co-infected patients in the present study also differed from the non co-infected individuals by geographical origin and ethnicity. It is possible that variation in host or viral genotype also influences HTLV-1 clonality. However, HTLV-1 genetic variation is limited, and the Cosmopolitan subtype 1a (Transcontinental subgroup) dominates in the Caribbean, South Africa, Peru and Brazil [31]). Moreover, the observed effects of host genotype account for only 5–10% of the observed variation of proviral load between individuals [32], [33], that is at least an order of magnitude less than the observed variation in proviral load within a given disease group.

The increased divergence between successive time-points in the HTLV-1 clonal composition observed in *Strongyloides* co-infected patients suggests a higher rate of persistent infectious spread of the virus, increasing the total number of clones generated during the HTLV-1 lifelong infection (as distinct from the total number of clones measured at a given time point). To test this hypothesis, further work is needed to quantify the rate of infectious spread within a given patient.

The observation that a major determinant of proviral load (in patients without ATLL) was the number of clones has important implications for the understanding of the development of HTLV-1-associated diseases. Why do some patients carry more clones than others? It is likely that the efficiency of the host's immune response to HTLV-1, especially the quality of the HTLV-1-specific CTLs [34], plays a major part in determining the total number of HTLV-1-infected clones. The initial dose of infection may also be a significant determinant. We also found that both IDH and *Strongyloides* co-infected patients had on average more abundant clones (i.e. the mean number of cells per clone was higher). These patients seem to be less capable of restricting clone abundance than those with HTLV-1 alone. Consistent with this conclusion, we observed a higher oligoclonality index in *Strongyloides* co-infected patients. Our observation of oligoclonal expansion in *Strongyloides* co-infected patients confirms a previous report by Gabet *et al* 2000 [16] but contrasts with the conclusion of polyclonal expansion made by Satoh *et al* 2002 [30]. The apparent discrepancy may be due to the lower sensitivity of the inverse long PCR technique used by Satoh *et al* [30] to quantify clonal distribution. Gabet *et al* also reported [35] oligoclonal expansion of HTLV-1^+^ T-cells in a patient with both IDH and *Strongyloides* co-infection, and the authors concluded that IDH might be a co-factor for ATLL. From our observations, it appears that *Strongyloides* co-infection could be a co-factor for oligoclonal expansion. However, in the samples from the IDH patients in this cohort we did not observe any case with a degree of oligoclonal proliferation (oligoclonality index value) within the ATLL range; and the oligoclonality index of HTLV-1-infected cells in the skin in IDH was significantly lower than that in the blood (see below).

Based on observations in a mouse model, it has been shown that immune activation of HTLV-1-infected CD4^+^ T-cells induces HTLV-1 Tax expression, T-cell proliferation, and may culminate in the development of ATLL [36]. Ratner *et al* [37] reported a case of a patient with HTLV-1-associated chronic ATLL and *Strongyloides* infection, in whom active HTLV-1 transcription resolved with anti-helminthic therapy [37]. This observation supports the idea that *Strongyloides* co-infection can induce HTLV-1 transcription via T-cell immune activation. Nevertheless, little is known about the nature of the hypothetical activating signals induced by the co-infection. We considered two possible explanations, which are not mutually exclusive: i. co-infection induces immune activation of the few infected clones that are specific to the co-infecting pathogen (such as *Strongyloides stercoralis*, *Staphylococcus* or *Streptococcus*); ii. co-infection favours expansion of all HTLV-1 infected clones (either by inducing non specific immune activation of the HTLV-1 infected clones or by impairing immune surveillance against the HTLV-1 infected cells). To answer this question we compared the HTLV-1 infected T-cells present in the skin lesion from IDH patients with the infected cells from the corresponding blood sample. As a reference point, we also compared the HTLV-1 infected T-cells present in the CSF in HAM/TSP patients with the infected cells from the corresponding blood sample. We observed that the major clone in the CSF can be rare or undetectable in the bloodstream (Figure S4 in Text S1). This supports the idea that certain infected clones present in the CSF can expand in the central nervous system (CNS), perhaps through antigenic stimulation; the relatively inefficient immune surveillance (the ‘immune privilege’) in the CNS may also allow unrestricted clonal expansion. By contrast, the presence of numerous HTLV-1 clones with an approximately equal abundance in the skin lesions of IDH patients suggests that the dermatitis does not involve the selective proliferation of HTLV-1 infected T-cells specific to *Staphylococcus* (or *Streptococcus*) antigens, but rather the non-specific proliferation of the entire population of infiltrating T-cells. HTLV-1 infection in endemic regions frequently occurs during breast-feeding and so predates infection with *Strongyloides* or *Streptococcus/Staphylococcus*. Consequently, it is unlikely that HTLV-1 infection is biased towards T-cells specific to *Strongyloides* or *Streptococcus*. It remains possible that chronic antigen stimulation favours the expansion of such T-cells in co-infected subjects. However, since the degree of oligoclonality of infected T-cells observed in patients with infective dermatitis was lower in the skin lesion than in blood, we infer that antigen specificity was a minor contributor to selective clonal expansion in these co-infected individuals. It remains possible that stimulation by *Strongyloides* antigens contributes to clonal expansion in individuals with *Strongyloides* co-infection. Our observation that the abundance of the largest clones in patients with *Strongyloides* co-infection or IDH is independent of the proviral insertion site environment supports the idea that *Strongyloides* co-infection and IDH change the selection forces that favour expansion of HTLV-1-infected clones.

HTLV-1 infection causes activation and proliferation of the infected T-cells. The HTLV-1 Tax protein activates the canonical NF-κB pathway (review by Qu *et al* [38]), upregulates expression of the interleukin-2 receptor alpha (IL-2Rα) [39] and promotes cell proliferation [40]. Additionally, the frequency of CD4^+^FoxP3^+^ regulatory T-cells is abnormally high in HTLV-1 patients [41] and the rate of CTL-mediated lysis of autologous HTLV-1-infected cells is negatively correlated with the frequency of CD4^+^FoxP3^+^ T-cells [41]. The frequency of CD4^+^FoxP3^+^ regulatory T-cells is increased further in *Strongyloides stercoralis* co-infected patients [15]. These observations suggest the existence of a vicious circle in which each pathogen favors the other. We suggest that two principal factors contribute to the increased clonal proliferation of HTLV-1^+^ T-cells observed in *Strongyloides* co-infection. First, an autocrine IL2/IL-2R loop was reported by Satoh *et al* [30] in patients with this co-infection. Second, the high frequency of CD4^+^FoxP3^+^ T-cells may impair the host T-cell response to HTLV-1 infection.

In summary, co-infection with *Strongyloides* is associated with an increase in the rate of formation of new HTLV-1-infected T-cell clones, oligoclonal proliferation of certain HTLV-1^+^ clones, and a higher mean clone abundance. IDH, similarly, is associated with an increase in the mean abundance of HTLV-1^+^ T-cells in the circulation and a change of the selection forces that favour expansion of HTLV-1^+^ clones. We propose that repeated activation of a large number of HTLV-1-infected T-cell clones causes abundant proviral expression, resulting in both infectious spread (infection of new T-cell clones) and mitotic spread (proliferation of existing infected T-cell clones) thereby increasing the risk of both inflammatory disease and malignant transformation.

## Materials and Methods

### Blood, skin lesion and cerebrospinal fluid samples

We studied 61 individuals (75% of Afrocaribbean ethnicity, 18% of Caucasian and 7% of Asian ethnicity) infected with HTLV-1 alone (14 asymptomatic HTLV-1 carriers, mean age 55 years; 1 patient with uveitis; 26 patients with HAM/TSP, mean age 62 years; 20 patients with ATLL, mean age 53 years). All individuals attended the clinic at the National Centre for Human Retrovirology (Imperial College Healthcare NHS Trust, St. Mary's Hospital, London, UK), and donated blood samples. Four HAM/TSP patients (2 from the UK, 2 from Brazil) also donated samples of cerebrospinal fluid (CSF). Fourteen individuals (11 from Peru, 3 Caribbean, mean age 44 years) infected with HTLV-1 and affected by strongyloidiasis donated blood samples. These patients had microbiology confirmed stool positive samples for *Strongyloides stercoralis* and confirmed negative post anti-helminth treatment. Seventeen individuals with IDH (10 from South Africa and 7 from Brazil, mean age 14 years) donated blood and skin lesion samples. These patients had active disease at time of blood sampling and biopsy. Table S1 in Text S1 details the microbiological isolates from a skin swab of each patient. PBMCs were isolated using Histopaque-1077 (Sigma-Aldrich). Cells were washed and cryopreserved in fetal calf serum (Invitrogen) with 10% DMSO (Sigma-Aldrich). Skin lesion samples were frozen directly after sampling in liquid nitrogen. DNA was extracted from PBMCs, skin tissue or CSF using DNeasy Blood and Tissue kit (Qiagen).

### Ethics statement

All subjects gave fully informed, written consent and all clinical investigations have been conducted according to the principles expressed in the Declaration of Helsinki. This study was approved by the UK National Research Ethics Service (NRES reference 09/H0606/106).

### HTLV-1 proviral load measurement

DNA was amplified for HTLV-1 DNA (using the Tax sequence-specific primers SK43 and SK44) and for β-actin (as a measure of genomic DNA using ActFw and ActRev primers) (SK43:5′CGGATACCCAGTCTACGTGT, SK44:5′GAGCCGATAACGCGTCCATCG, ActFw:5′TCACCCACACTGTGCCCATCTATGA, ActRev:5′CATCGGAACCGCTCATTGCCGATAG). Three dilutions of DNA were amplified by real time quantitative PCR in a Roche light cycler using SYBR Green 1 Dye incorporation (Roche Applied Science). Standard curves were generated using the rat cell line TARL2 which contains 1 copy per cell of the HTLV-1 provirus [42]. The sample copy number was estimated by interpolation from the standard curve, calculated as an average of the 3 dilutions, and expressed as the number of copies per 10^6^ PBMCs.

### Selective amplification and quantification of proviral insertion sites

We used a newly developed protocol to map and quantify thousands of HTLV-1 proviral insertion sites, as previously described [7], [43]. We define an HTLV-1 clone as a population of cells that carry an integrated HTLV-1 provirus in a particular insertion site in the host genome. We have demonstrated that there is a single proviral copy per cell in non-transformed cells naturally infected with HTLV-1 [44], and leukemic clones typically carry one (complete or defective) provirus per cell [45] [46]. DNA was extracted from uncultured PBMCs, skin lesion or CSF of HTLV-1-infected individuals and sheared by sonication. A partially double-stranded DNA linker containing a 6 nt index tag was ligated to the sheared DNA and nested PCR was performed between the HTLV-1 LTR and the linker. Nested PCR products were pooled to construct the library of DNA for high-throughput sequencing. Fifty-nucleotide paired-end reads (read 1 and read 2) and a 6 nucleotide index tag read were acquired on an Illumina Genome Analyzer II or an Illumina HiSeq. Read 1 and read 2 were mapped against the human genome (build hg18) and the proviral insertion site and the shear site were deduced. For each unique insertion site, we counted the number of amplicons of different length (i.e. different shear sites) to enumerate the number of sister cells of that infected T-cell clone. The absolute abundance of a given clone *i* (number of cells per 10^6^ PBMCs) was calculated from the number of sister cells and the measurement of the proviral load as follow:
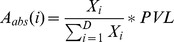
where *X_i_* is the number of sister cells of the *i*th clone, *D* the number of observed clones and *PVL* the proviral load.

The relative abundance of a given clone *i* (in percent of the proviral load) was expressed as follow:
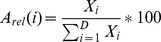



### Oligoclonality index

To measure the clonality of the infected cell population, i.e. the non-uniformity of the clone abundance distribution, we used the oligoclonality index [7], based on the Gini coefficient [47].

Oligoclonality index = 1 indicates perfect monoclonality (only one clone constitutes the total proviral load), while oligoclonality index = 0 indicates perfect polyclonality (all clones have the same abundance).

### Similarity indices

Quantitative measures of similarity (or overlap) between two populations play an important role in statistical ecology. The first similarity indices developed were based on the presence or absence of species between the two populations. The widely used SØrensen incidence-based similarity index ranges from 0 to 1, with 0 indicating that no clones are shared between the two populations and 1 indicating complete identity (all the clones present in population 1 were also present in population 2 and *vice versa*). The former index was subsequently improved to take into consideration the abundance of each clone and named SØrensen abundance-based similarity index. Because this index takes clone abundance into account, populations that contain the same clones but have different clone abundance will have an index value of less than 1. Details of the calculations are given in supplemental data.

In Figure 2, the similarity index was calculated in each case by comparing two samples of equal numbers of sister cells, to preclude a bias toward the clone distribution in one sample. When samples from 3 time points for a given patient were analysed, we calculated the similarity index twice, between time point 1 and time point 2 and between time point 2 and time point 3 respectively. In Figure 4, to compare blood and skin clone populations or blood and CSF clone populations from the same patient, we first created subsamples of clones from the blood clonality analysis by randomly drawing 10% of the sister cells detected in the blood. We calculated the similarity index by comparing 2 different subsamples from the blood population (BLD vs BLD values). We then created a subsample of clones from the blood by randomly drawing the same number of sister cells detected in the corresponding skin lesion (or CSF) and calculated the similarity index between the blood subsample and the skin (or CSF) (BLD vs SKN or BLD vs CSF respectively).

### Genetic and epigenetic environment around the proviral insertion site

We used the Integration Site Pipeline and Database (INSIPID) from the Bushman laboratory (Department of Microbiology, University of Pennsylvania School of Medicine, Pennsylvania, Philadelphia, United States of America) (http://microb215.med.upenn.edu/insipid/). This web-based tool houses sequences of newly inserted elements in vertebrate genomes, together with specific genomic annotations, to facilitate analysis of the environment of the genomic insertion site: see Figure S3 in Text S1.

### Total number of clones in the blood and average clone abundance in the blood

The diversity estimation approach (DivE) (Daniel Laydon, Charles Bangham, Becca Asquith, submitted) used to estimate the total number of clones (observed and unobserved) involves fitting many mathematical models to species-accumulation data, and to successively smaller nested subsamples thereof. Novel criteria are used to score models in how consistently they can reproduce existing observations from incomplete data. The estimates from the best performing models are aggregated (using the geometric mean) to estimate the number of clones in the circulation. We have shown that, when applied to HTLV-1 infection and other microbiological populations, DivE significantly outperforms several classical ecological estimators of unseen species (namely the Chao, Bootstrap and Good-Turing estimators, and species-accumulation curves). Let 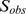 be the total number of clones observed, and let 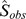 be an estimate of 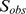 from a subsample of data. We define the accuracy of a given estimator as the percentage error between 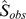 and 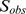 (i.e. 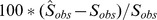). When applied to HTLV-1 infection, the mean accuracy of DivE was 3.5%, compared to accuracies of 61.5%, 35.3%, 35.0%, and 29.1% for the Chao, Bootstrap and Good-Turing estimators, and the species-accumulation curves respectively (using two-tailed paired Mann-Whitney tests, p<0.0001 for all comparisons with DivE). DivE was optimized for clonal distribution of patients with non-malignant HTLV-1 infection and further work will be necessary to estimate with the same confidence the total number of clones in ATLL patients. Therefore, within this paper we do not estimate the total number of clones in patients with ATLL.

The average clone abundance was calculated from the proviral load divided by the estimated total number of clones in the blood and expressed as the number of cells per 10^6^ PBMCs.
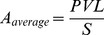
where *PVL* is the proviral load and *S* the estimated total number of clones.

### Statistics

Statistical tests were performed using GraphPad Prism and R softwares and were two tailed when possible. The symbol *** was used when p<0.001, ** when p<0.01, * when p<0.05, NS (Non Significant) when p>0.05.

### Accession numbers (RefSeqGene)

CXCR4, NG_011587.1; CCR5, NG_012637.1; IL-2Rα, NG_007403.1; IFN-γ, NG_015840.1; IL-10, NG_012088.1; TGF-β, NG_013364.1; HTLV-1 Tax, NC_001436.1.

## Supporting Information

Text S1 The supporting file Text S1 contains supporting figures S1, S2, S3 and S4, supporting methods for similarity indices calculation and supporting tables S1 and S2.
(PDF)

## References

[1] Proietti FA, Carneiro-Proietti AB, Catalan-Soares BC, Murphy EL (2005) Global epidemiology of HTLV-I infection and associated diseases. Oncogene 24: 6058–6068. 16155612 10.1038/sj.onc.1208968

[2] Kubota R, Fujiyoshi T, Izumo S, Yashiki S, Maruyama I, et al (1993) Fluctuation of HTLV-I proviral DNA in peripheral blood mononuclear cells of HTLV-I-associated myelopathy. J Neuroimmunol 42: 147–154. 8429100 10.1016/0165-5728(93)90004-i

[3] Nagai M, Usuku K, Matsumoto W, Kodama D, Takenouchi N, et al (1998) Analysis of HTLV-I proviral load in 202 HAM/TSP patients and 243 asymptomatic HTLV-I carriers: high proviral load strongly predisposes to HAM/TSP. J Neurovirol 4: 586–593. 10065900 10.3109/13550289809114225

[4] Etoh K, Yamaguchi K, Tokudome S, Watanabe T, Okayama A, et al (1999) Rapid quantification of HTLV-I provirus load: detection of monoclonal proliferation of HTLV-I-infected cells among blood donors. Int J Cancer 81: 859–864. 10362130 10.1002/(sici)1097-0215(19990611)81:6<859::aid-ijc4>3.0.co;2-k

[5] Nejmeddine M, Bangham CR (2010) The HTLV-1 Virological Synapse. Viruses 2: 1427–1447. 21994688 10.3390/v2071427 PMC3185711

[6] Bangham CR, Osame M (2005) Cellular immune response to HTLV-1. Oncogene 24: 6035–6046. 16155610 10.1038/sj.onc.1208970

[7] Gillet NA, Malani N, Melamed A, Gormley N, Carter R, et al (2011) The host genomic environment of the provirus determines the abundance of HTLV-1-infected T-cell clones. Blood 117: 3113–3122. 21228324 10.1182/blood-2010-10-312926 PMC3062313

[8] Marcos LA, Terashima A, Dupont HL, Gotuzzo E (2008) Strongyloides hyperinfection syndrome: an emerging global infectious disease. Trans R Soc Trop Med Hyg 102: 314–318. 18321548 10.1016/j.trstmh.2008.01.020

[9] Robinson RD, Lindo JF, Neva FA, Gam AA, Vogel P, et al (1994) Immunoepidemiologic studies of Strongyloides stercoralis and human T lymphotropic virus type I infections in Jamaica. J Infect Dis 169: 692–696. 8158055 10.1093/infdis/169.3.692

[10] Gotuzzo E, Terashima A, Alvarez H, Tello R, Infante R, et al (1999) Strongyloides stercoralis hyperinfection associated with human T cell lymphotropic virus type-1 infection in Peru. Am J Trop Med Hyg 60: 146–149. 9988339 10.4269/ajtmh.1999.60.146

[11] Courouble G, Rouet F, Hermann-Storck C, Nicolas M, Candolfi E, et al (2000) Human T-cell lymphotropic virus Type I association with Strongyloides stercoralis: a case control study among Caribbean blood donors from Guadeloupe (French West Indies). J Clin Microbiol 38: 3903–3904. 11184174 10.1128/jcm.38.10.3903-3904.2000 PMC87508

[12] Terashima A, Alvarez H, Tello R, Infante R, Freedman DO, et al (2002) Treatment failure in intestinal strongyloidiasis: an indicator of HTLV-I infection. Int J Infect Dis 6: 28–30. 12044298 10.1016/s1201-9712(02)90132-3

[13] Satoh M, Toma H, Sato Y, Takara M, Shiroma Y, et al (2002) Reduced efficacy of treatment of strongyloidiasis in HTLV-I carriers related to enhanced expression of IFN-gamma and TGF-beta1. Clin Exp Immunol 127: 354–359. 11876761 10.1046/j.1365-2249.2002.01733.x PMC1906331

[14] Hirata T, Uchima N, Kishimoto K, Zaha O, Kinjo N, et al (2006) Impairment of host immune response against strongyloides stercoralis by human T cell lymphotropic virus type 1 infection. Am J Trop Med Hyg 74: 246–249. 16474078

[15] Montes M, Sanchez C, Verdonck K, Lake JE, Gonzalez E, et al (2009) Regulatory T cell expansion in HTLV-1 and strongyloidiasis co-infection is associated with reduced IL-5 responses to Strongyloides stercoralis antigen. PLoS Negl Trop Dis 3: e456. 19513105 10.1371/journal.pntd.0000456 PMC2686100

[16] Gabet AS, Mortreux F, Talarmin A, Plumelle Y, Leclercq I, et al (2000) High circulating proviral load with oligoclonal expansion of HTLV-1 bearing T cells in HTLV-1 carriers with strongyloidiasis. Oncogene 19: 4954–4960. 11042682 10.1038/sj.onc.1203870

[17] Pagliuca A, Layton DM, Allen S, Mufti GJ (1988) Hyperinfection with strongyloides after treatment for adult T cell leukaemia-lymphoma in an African immigrant. BMJ 297: 1456–1457. 3147015 10.1136/bmj.297.6661.1456 PMC1835153

[18] Massey AC, Weinstein DL, Petri WA, Williams ME, Hess CE (1990) ATLL complicated by strongyloidiasis and isosporiasis: case report. Va Med Q 117: 311–316. 2375179

[19] Phelps KR (1993) Strongyloides hyperinfection in patients coinfected with HTLV-I and S. stercoralis. Am J Med 94: 447–449. 8475941 10.1016/0002-9343(93)90163-j

[20] Phelps KR, Ginsberg SS, Cunningham AW, Tschachler E, Dosik H (1991) Case report: adult T-cell leukemia/lymphoma associated with recurrent strongyloides hyperinfection. Am J Med Sci 302: 224–228. 1928233 10.1097/00000441-199110000-00006

[21] Plumelle Y, Gonin C, Edouard A, Bucher BJ, Thomas L, et al (1997) Effect of Strongyloides stercoralis infection and eosinophilia on age at onset and prognosis of adult T-cell leukemia. Am J Clin Pathol 107: 81–87. 8980372 10.1093/ajcp/107.1.81

[22] Plumelle Y, Pascaline N, Nguyen D, Panelatti G, Jouannelle A, et al (1993) Adult T-cell leukemia-lymphoma: a clinico-pathologic study of twenty-six patients from Martinique. Hematol Pathol 7: 251–262. 8113152

[23] Agape P, Copin MC, Cavrois M, Panelatti G, Plumelle Y, et al (1999) Implication of HTLV-I infection, strongyloidiasis, and P53 overexpression in the development, response to treatment, and evolution of non-Hodgkin's lymphomas in an endemic area (Martinique, French West Indies). J Acquir Immune Defic Syndr Hum Retrovirol 20: 394–402. 10096585 10.1097/00042560-199904010-00011

[24] Pagliuca A (1999) Strongyloides hyperinfection in adult T-cell leukaemia/lymphoma. Br J Haematol 105: 1. 10.1111/j.1365-2141.1999.01401.x 10366242

[25] Lee R, Schwartz RA (2011) Human T-lymphotrophic virus type 1-associated infective dermatitis: a comprehensive review. J Am Acad Dermatol 64: 152–160. 20691499 10.1016/j.jaad.2009.10.021

[26] Primo J, Siqueira I, Nascimento MC, Oliveira MF, Farre L, et al (2009) High HTLV-1 proviral load, a marker for HTLV-1 associated myelopathy/tropical spastic paraparesis, is also detected in patients with infective dermatitis associated with HTLV-1. Braz J Med Biol Res 42: 761–764. 19578703 10.1590/s0100-879x2009005000008 PMC2963476

[27] de Oliveira Mde F, Bittencourt AL, Brites C, Soares G, Hermes C, et al (2004) HTLV-I associated myelopathy/tropical spastic paraparesis in a 7-year-old boy associated with infective dermatitis. J Neurol Sci 222: 35–38. 15240193 10.1016/j.jns.2004.04.006

[28] Nascimento MC, Primo J, Bittencourt A, Siqueira I, de Fatima Oliveira M, et al (2009) Infective dermatitis has similar immunological features to human T lymphotropic virus-type 1-associated myelopathy/tropical spastic paraparesis. Clin Exp Immunol 156: 455–462. 19438598 10.1111/j.1365-2249.2008.03869.x PMC2691974

[29] de Oliveira Mde F, Vieira MG, Primo J, Siqueira IC, Carvalho EM, et al (2010) Flower cells in patients with infective dermatitis associated with HTLV-1. J Clin Virol 48: 288–290. 20541459 10.1016/j.jcv.2010.05.005

[30] Satoh M, Toma H, Sugahara K, Etoh K, Shiroma Y, et al (2002) Involvement of IL-2/IL-2R system activation by parasite antigen in polyclonal expansion of CD4(+)25(+) HTLV-1-infected T-cells in human carriers of both HTLV-1 and S. stercoralis. Oncogene 21: 2466–2475. 11971181 10.1038/sj.onc.1205329

[31] Gessain A, Cassar O (2012) Epidemiological Aspects and World Distribution of HTLV-1 Infection. Front Microbiol 3: 388. 23162541 10.3389/fmicb.2012.00388 PMC3498738

[32] Vine AM, Witkover AD, Lloyd AL, Jeffery KJ, Siddiqui A, et al (2002) Polygenic control of human T lymphotropic virus type I (HTLV-I) provirus load and the risk of HTLV-I-associated myelopathy/tropical spastic paraparesis. J Infect Dis 186: 932–939. 12232833 10.1086/342953

[33] Jeffery KJ, Usuku K, Hall SE, Matsumoto W, Taylor GP, et al (1999) HLA alleles determine human T-lymphotropic virus-I (HTLV-I) proviral load and the risk of HTLV-I-associated myelopathy. Proc Natl Acad Sci U S A 96: 3848–3853. 10097126 10.1073/pnas.96.7.3848 PMC22383

[34] Bangham CR (2009) CTL quality and the control of human retroviral infections. Eur J Immunol 39: 1700–1712. 19582737 10.1002/eji.200939451

[35] Gabet AS, Kazanji M, Couppie P, Clity E, Pouliquen JF, et al (2003) Adult T-cell leukaemia/lymphoma-like human T-cell leukaemia virus-1 replication in infective dermatitis. Br J Haematol 123: 406–412. 14616998 10.1046/j.1365-2141.2003.04565.x

[36] Swaims AY, Khani F, Zhang Y, Roberts AI, Devadas S, et al (2010) Immune activation induces immortalization of HTLV-1 LTR-Tax transgenic CD4+ T cells. Blood 116: 2994–3003. 20634377 10.1182/blood-2009-07-231050 PMC2974607

[37] Ratner L, Grant C, Zimmerman B, Fritz J, Weil G, et al (2007) Effect of treatment of Strongyloides infection on HTLV-1 expression in a patient with adult T-cell leukemia. Am J Hematol 82: 929–931. 17617788 10.1002/ajh.20929 PMC2652703

[38] Qu Z, Xiao G (2011) Human T-cell lymphotropic virus: a model of NF-kappaB-associated tumorigenesis. Viruses 3: 714–749. 21743832 10.3390/v3060714 PMC3131208

[39] Ballard DW, Bohnlein E, Lowenthal JW, Wano Y, Franza BR, et al (1988) HTLV-I tax induces cellular proteins that activate the kappa B element in the IL-2 receptor alpha gene. Science 241: 1652–1655. 2843985 10.1126/science.241.4873.1652

[40] Asquith B, Zhang Y, Mosley AJ, de Lara CM, Wallace DL, et al (2007) In vivo T lymphocyte dynamics in humans and the impact of human T-lymphotropic virus 1 infection. Proc Natl Acad Sci U S A 104: 8035–8040. 17483473 10.1073/pnas.0608832104 PMC1861853

[41] Toulza F, Heaps A, Tanaka Y, Taylor GP, Bangham CR (2008) High frequency of CD4+FoxP3+ cells in HTLV-1 infection: inverse correlation with HTLV-1-specific CTL response. Blood 111: 5047–5053. 18094326 10.1182/blood-2007-10-118539 PMC2602587

[42] Tateno M, Kondo N, Itoh T, Chubachi T, Togashi T, et al (1984) Rat lymphoid cell lines with human T cell leukemia virus production. I. Biological and serological characterization. J Exp Med 159: 1105–1116. 6323614 10.1084/jem.159.4.1105 PMC2187267

[43] Berry CC, Gillet NA, Melamed A, Gormley N, Bangham CR, et al (2012) Estimating abundances of retroviral insertion sites from DNA fragment length data. Bioinformatics 28: 755–762. 22238265 10.1093/bioinformatics/bts004 PMC3307109

[44] Cook LB, Rowan AG, Melamed A, Taylor GP, Bangham CR (2012) HTLV-1-infected T cells contain a single integrated provirus in natural infection. Blood 120: 3488–90. 22955925 10.1182/blood-2012-07-445593 PMC3482858

[45] Tamiya S, Matsuoka M, Etoh K, Watanabe T, Kamihira S, et al (1996) Two types of defective human T-lymphotropic virus type I provirus in adult T-cell leukemia. Blood 88: 3065–3073. 8874205

[46] Ohshima K, Ohgami A, Matsuoka M, Etoh K, Utsunomiya A, et al (1998) Random integration of HTLV-1 provirus: increasing chromosomal instability. Cancer Lett 132: 203–212. 10397475 10.1016/s0304-3835(98)00188-8

[47] Gini C (1914) Sulla misura della concentrazione e della variabilita dei caratteri: Transactions of the Real Istituto Veneto di Scienze. Ferrari

