# Supplementary material for: Strongyloidiasis and Infective Dermatitis Alter Human T Lymphotropic Virus-1 Clonality in vivo
Source: PLoS Pathog. 2013 Apr 4;9(4):e1003263. doi: 10.1371/journal.ppat.1003263 (PMC3617147; doi:10.1371/journal.ppat.1003263)
Supplement: Text S1 — The supporting file Text S1 contains supporting figures S1, S2, S3 and S4, supporting methods for similarity indices calculation and supporting tables S1 and S2. (PDF) [file ppat.1003263.s001.pdf]

Figure S1

A. HTLV-1 clone abundance distribution in the blood

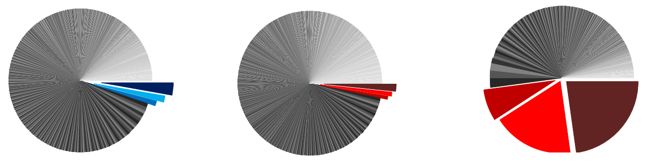

	Infective dermatitis (IDH)	*Strongyloides stercoralis* (St)	
Oligoclonality index	0.48	0.39	0.76
Number of unique insertion sites detected	1,285	1,864	626
Proviral load (copies per 10^6 PBMCs)	$9.9*10^4$	$1.9*10^5$	$1.4*10^5$

B. Oligoclonality index evolution in *Strongyloides* co-infected patients

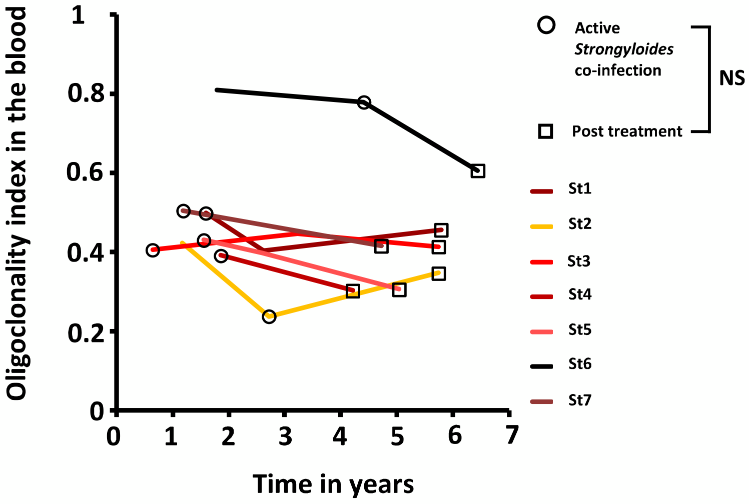

Figure S1: A. Clonal distribution in genomic DNA from peripheral blood mononuclear cells of representative subjects. The size of each slice in the pie-chart represents the relative abundance of each respective proviral insertion site in that subject. The three most abundant clones are colored. **B.** Anti-helminth treatment did not significantly change the oligoclonality index in *Strongyloides* co-infected patients (paired t-test).

Figure S2

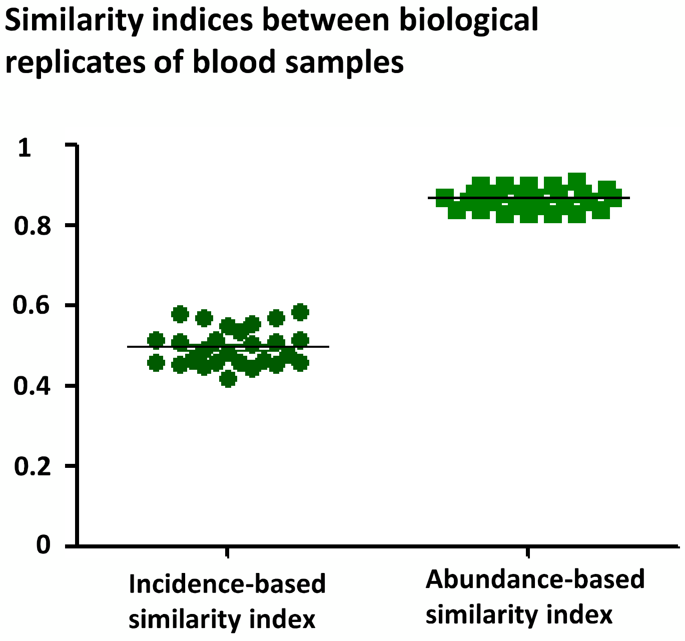

Figure S2: Incidence-based and abundance-based similarity index calculated by comparing two clonality analyses made from the same blood sample in patients infected with HTLV-1 only.

Figure S3

Data generated by our high-throughput sequencing technique and used as input for INSIPID

Output from INSIPID

Patients with HTLV-1 only, leukemic clones

Patient code	Disease status	Chromosome	Position	Strand	Clone abundance (no of cells per 10^6 PBMCs)	inGene (Refseq)	inGene Ort	Nearest gene distance (bp)	Nearest CpG island distance (bp)	H2BK120 ac Count 10k	H2BK20 ac Count 10k	H2BK5 ac Count 10k	H3K18 ac Count 10k	H3K27 ac Count 10k	H3K4ac Count 10k	H3K9 me2 Count 10k	H3K9 me3 Count 10k	H4K20 me3 Count 10k	H4K5ac Count 10k	H4K8ac Count 10k	H4K91ac Count 10k
C3	chronic ATL	chr5	94400662	+	6.4E+05	MCTP1	-	42639	244553	1	4	3	2	1	0	33	38	10	4	7	2
LEU	lymphoma	chr1	20877564	+	4.2E+05	KIF17	-	-14471	-16739	7	4	6	16	8	6	12	15	12	18	24	5
JH	acute ATL	chr2	138170969	+	3.1E+05	FALSE	NA	-19212	804534	3	5	6	2	1	2	55	24	17	4	4	2
LEP	acute ATL	chr3	197773499	-	2.0E+05	WDR53	-	-6311	-6236	35	52	41	28	43	30	21	3	4	49	29	37
C4	chronic ATL	chr7	90098693	-	2.0E+05	FALSE	NA	-77954	34393	6	1	5	1	4	1	28	15	7	1	5	2
AN	acute ATL	chr9	112538958	+	1.9E+05	MUSK	+	64141	-157108	1	3	3	6	4	2	58	32	24	4	2	1
LEZ	acute ATL	chr12	129441003	+	1.8E+05	FALSE	NA	5630	33727	3	3	8	2	8	4	58	15	65	6	11	3
KD5	acute ATL	chr9	15797049	+	1.5E+05	C9orf93	+	164848	-253715	2	1	2	6	2	2	44	28	8	1	3	0
HCG-LEY	chronic ATL	chrX	147227167	-	1.5E+05	FALSE	NA	-162663	-162386	68	56	56	77	65	61	27	11	29	29	40	60
S1	smouldering ATL	chr13	35811059	-	1.3E+05	SPG20	-	-7587	-6678	14	18	6	9	7	11	42	9	8	13	22	7
S6	smouldering ATL	chr8	142483050	-	1.2E+05	FALSE	NA	-18138	11374	194	181	194	219	184	182	12	4	13	141	98	267
S4	smouldering ATL	chr1	182126012	+	9.2E+04	RGL1	+	38277	-84973	60	60	28	84	55	52	36	7	11	67	51	37
S2	smouldering ATL	chr10	51234264	+	3.6E+04	FALSE	NA	849	813	66	61	82	83	91	52	25	9	12	54	71	79
S5	smouldering ATL	chr16	64689163	+	3.5E+04	FALSE	NA	268862	172623	2	3	6	5	3	13	50	14	10	5	7	2
TBX-LFI	lymphoma	chr1	151510970	+	8.5E+03	FALSE	NA	-9746	264078	4	7	3	6	2	5	30	20	10	7	5	5
LFC	chronic ATL	chr13	45858440	-	8.5E+03	13orf18	-	-1196	-245	13	26	16	36	19	19	28	12	11	31	36	28
LFA	cutaneous ATL	chr15	36147866	-	6.6E+03	FALSE	NA	116951	-4421	20	18	7	21	6	19	55	35	15	26	24	12
P7	chronic ATL	chr11	31332995	-	3.8E+03	DCDC1	-	-14902	-14668	8	9	3	8	3	4	13	15	4	9	8	1
LFE	lymphoma	chr5	60952642	-	1.9E+03	FALSE	NA	-16750	-4649	8	12	5	7	1	11	37	12	18	12	12	5

6 clones out of 19 lie within +/- 10kb of a gene: Pr=32% Pr random=19%

6 clones out of 19 lie within +/- 10kb of a CpG island: Pr=32% Pr random=15%

The mean count N=24 N random=15

Figure S3: Calculation of the values shown in Figure 3. The first 6 columns are data used as input for INSIPID. For each clone (each row) the following are given: patient code, disease status, position and orientation of the provirus (Chromosome, Position and Strand columns), and clone abundance.
The other columns are the output from INSIPID:
"In Gene" column gives the name of the Gene when the provirus is inserted inside it, or FALSE if not. Here, 'gene' refers to RefSeq genes.
"inGene Orientation " column gives the orientation (+ or – strand) of the gene when applicable
"nearest Gene Distance (bp)" column gives the distance in bp to the nearest gene (3' or 5' end)
"nearest CpG Island Distance (bp)" column gives the distance to the nearest CpG island
The columns "histoneX Count 10k" give the number of that particular histone mark in a 10kb window around the proviral insertion site. These data come from 2 resource papers [1,2].

Figure S4

Clonal distribution in blood and cerebro-spinal fluid of HAM/TSP patients

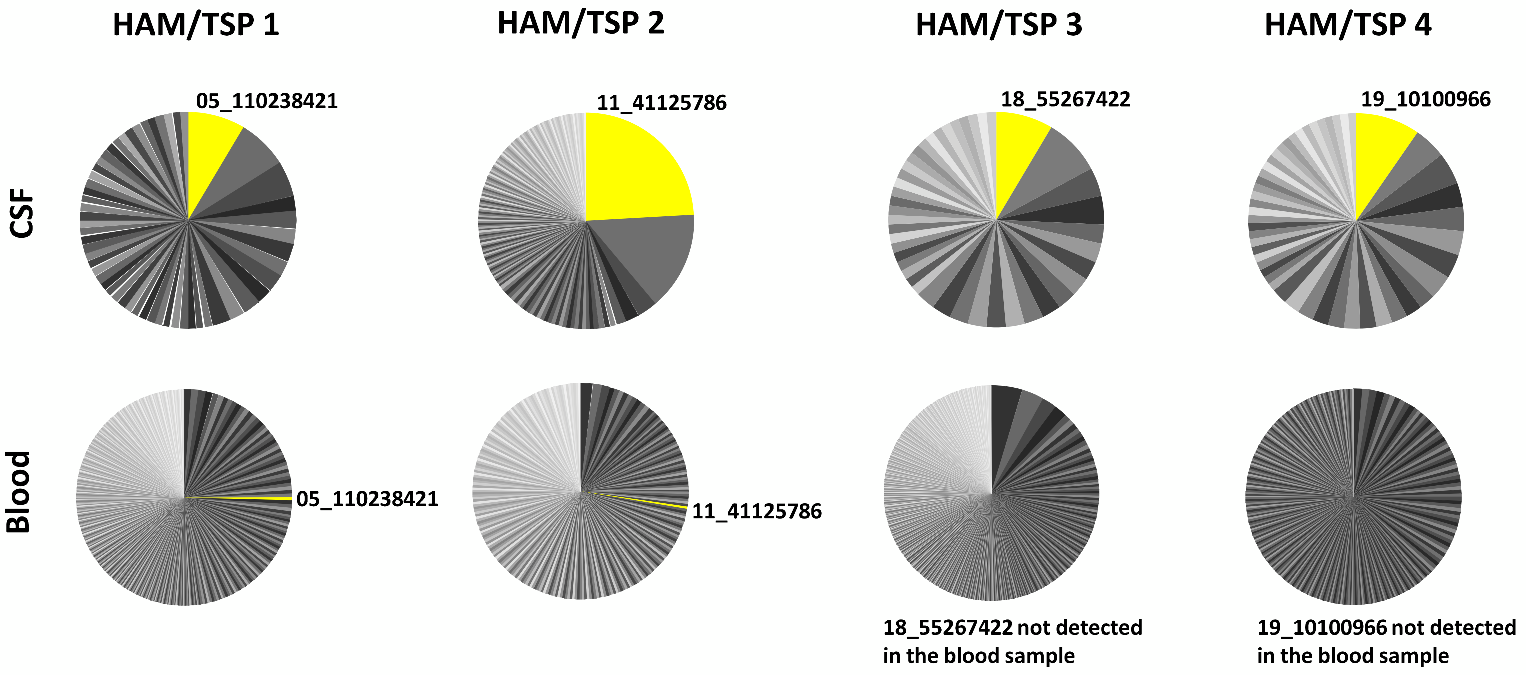

Figure S4: Clonal distribution in CSF and blood corresponding samples from 4 HAM/TSP patients. The yellow colored slice represents the most abundant clone in the CSF.

Supporting methods

Similarity indices

SØrensen incidence-based similarity index is defined as

$$S_i = \frac{2D_{12}}{D_1 + D_2}$$

where

D_1 : number of observed clones in population 1

D_2 : number of observed clones in population 2

D_{12} : number of observed shared clones in the two populations

SØrensen abundance-based similarity index is defined as

$$S_a = \frac{2UV}{U + V}$$

where

(X_i, Y_i) : number of sister cells of the ith clone in the two populations

$$n = \sum_{i=1}^{D_1} X_i$$

$$m = \sum_{i=1}^{D_2} Y_i$$

f_{1+} : the observed number of shared clones that occur once in population 1 (these clones must be present in population 2, but may have any frequency)

f_{2+} : the observed number of shared clones that occur twice in population 1

f_{+1} : the observed number of shared clones that occur once in population 2 (these clones must be present in population 1, but may have any frequency)

f_{+2} : the observed number of shared clones that occur twice in population 2

$$U = \sum_{i=1}^{D_{12}} \frac{X_i}{n} + \frac{(m-1)}{m} \frac{f_{+1}}{2f_{+2}} \sum_{i=1}^{D_{12}} \frac{X_i}{n} I(Y_i = 1)$$

$$V = \sum_{i=1}^{D_{12}} \frac{Y_i}{m} + \frac{(n-1)}{n} \frac{f_{1+}}{2f_{2+}} \sum_{i=1}^{D_{12}} \frac{Y_i}{m} I(X_i = 1)$$

Supporting tables

Table S1: Characteristics of patients with HTLV-1 associated infective dermatitis

Patient code	Country of origin	Gender	Age at presentation , years	Age at biopsy/blood test, years	Skin swab microbiological isolates
**IDH1**	South Africa	Female	15	23	*Staphylococcal aureus*
**IDH2**	South Africa	Male	8	11	No organsims cultured
**IDH3**	South Africa	Female	7	11	No organisms cultured
**IDH4**	South Africa	Female	8	13	*Staphylococcal aureus*
**IDH5**	South Africa	Female	8	12	*Staphylococcal aureus* and *Streptococcus pyogenes*
**IDH6**	South Africa	Male	7	12	No organisms cultured
**IDH7**	South Africa	Male	8	15	*Staphylococcal aureus*
**IDH8**	South Africa	Male	11	17	*Staphylococcal aureus* and *beta haemolytic Streptococcus*
**DH9**	South Africa	Male	8	8	*Staphylococcal aureus*
**IDH10**	South Africa	Female	15	20	*Staphylococcal aureus*
**IDH11**	Brazil	Female	2	13	*Staphylococcal aureus*
**IDH12**	Brazil	Male	4	16	*Staphylococcal aureus*
**IDH13**	Brazil	Female	2	18	none collected
**IDH14**	Brazil	Male	0.9	12	*Staphylococcal aureus*
**IDH15**	Brazil	Male	1	16	none collected
**IDH16**	Brazil	Female	1	3	*Staphylococcal aureus*
**IDH17**	Brazil	Female	2	10	*Staphylococcal aureus*
**IDH18***	Brazil	Female	0.7	22	*Staphylococcal aureus*
**IDH19***	Brazil	Female	6	9	*Staphylococcal aureus*

*IDH18 and IDH19 patients both diagnosed with HAM/TSP in addition to IDH.

Table S2: ***Strongyloides*** **co-infection status of HTLV-1 patients at different time points before and after anti-helminth treatment.**

Patient code	Country of origin	DOB	Date of blood test	Gender	Age at strongyloidiasis diagnosis	Age at blood test	Strongyloides 'Active' or 'post treatment'
**St2**	Peru	4/07/1961	3/08/2005	Female	44	44	**active**
			16/08/2006			45	**active**
			13/10/2009			48	**cleared**
**St3**	Peru	15/08/1944	1/03/2005	Female	60	60	**active**
			18/09/2006			62	**active**
			26/09/2009			65	**cleared**
**St4**	Peru	2/03/1963	21/08/2004	Female	41	41	**active**
			21/03/2007			44	**active**
			26/09/2009			46	**cleared**
**St6**	Peru	11/07/1983	9/11/2005	Male	22	22	**active**
			17/03/2008			24	**cleared**
**St7**	Peru	28/02/1964	21/07/2005	Female	41	41	**active**
			3/08/2006			42	**active**
			13/01/2009			44	**cleared**
**St9**	Peru	24/03/1954	12/10/2005	Male	51	51	**unconfirmed -presumed infected**
			28/05/2008			54	**active**
			9/06/2010			56	**cleared**
**St11**	Peru	11/09/1960	9/03/2005	Female	44	44	**unconfirmed -presumed infected**
			18/09/2008			48	**none**

References

1. Barski A, Cuddapah S, Cui K, Roh TY, Schones DE, et al. (2007) High-resolution profiling of histone methylations in the human genome. Cell 129: 823-837.
2. Wang Z, Zang C, Rosenfeld JA, Schones DE, Barski A, et al. (2008) Combinatorial patterns of histone acetylations and methylations in the human genome. Nat Genet 40: 897-903.
